# Nanostructured plasmonic substrates for use as SERS sensors

**DOI:** 10.1186/s40580-016-0078-6

**Published:** 2016-08-01

**Authors:** Tae Yoon Jeon, Dong Jae Kim, Sung-Gyu Park, Shin-Hyun Kim, Dong-Ho Kim

**Affiliations:** 1 Department of Chemical and Biomolecular Engineering (BK21+ Program), KAIST, Daejeon, 305-701 Republic of Korea; 2 Advanced Functional Thin Films Department, Korea Institute of Materials Science (KIMS), Changwon, Gyeongnam 641-831 Republic of Korea

**Keywords:** LSPR (localized surface plasmon resonance), SERS (surface-enhanced Raman scattering), Nanobiosensor, Nanogap, Nanolithography, Self-assembly

## Abstract

Plasmonic nanostructures strongly localize electric fields on their surfaces via the collective oscillations of conducting electrons under stimulation by incident light at a certain wavelength. Molecules adsorbed onto the surfaces of plasmonic structures experience a strongly enhanced electric field due to the localized surface plasmon resonance (LSPR), which amplifies the Raman scattering signal obtained from these adsorbed molecules. This phenomenon is referred to as surface-enhanced Raman scattering (SERS). Because Raman spectra serve as molecular fingerprints, SERS has been intensively studied for its ability to facilely detect molecules and provide a chemical analysis of a solution. Further enhancements in the Raman intensity and therefore higher sensitivity in SERS-based molecular analysis have been achieved by designing plasmonic nanostructures with a controlled size, shape, composition, and arrangement. This review paper focuses on the current state of the art in the fabrication of SERS-active substrates and their use as chemical and biosensors. Starting with a brief description of the basic principles underlying LSPR and SERS, we discuss three distinct nanofabrication methods, including the bottom-up assembly of nanoparticles, top-down nanolithography, and lithography-free random nanoarray formation. Finally, typical applications of SERS-based sensors are discussed, along with their perspectives and challenges.

## Introduction

### Surface-enhanced Raman scattering (SERS)

Raman scattering is the inelastic scattering of a photon as a result of the interaction between light and a molecule [1]. Most of the scattered photons have the same energy as the incident photons (i.e., Rayleigh scatting), but a small fraction of the scattered photons lose or gain certain amounts of energy due to energy exchange between the scattering partners. Those changes in energy (or frequency shift) are related to the characteristic energies of the vibrational or rotational modes of a molecule. Due to the high selectivity of Raman scattering, the Raman signal can be used as a molecular fingerprint for unknown molecules [2, 3]. Raman scattering spectra are typically measured using monochromatic light source (usually a laser) to excite the molecular energy levels, and scattered light is detected after interacting with the molecules of interest. Scattered photons that lose energy are called Stokes scattering, and anti-Stokes scattering corresponds to photons that gain energy relative to the energy of the incident photon. The magnitude of the energy shift is called the Raman shift and can be expressed by

1 $$\Delta \omega \left( {{\text{cm}}^{ - 1} } \right) = \left( {\frac{1}{{\lambda_{0} \left( {\text{nm}} \right)}} - \frac{1}{{\lambda_{1} \left( {\text{nm}} \right)}}} \right) \times \frac{{\left( {10^{7} {\text{nm}}} \right)}}{{\left( {\text{cm}} \right)}},$$

where *λ*_0_ and *λ*_1_ are the wavelengths of the incident and Raman scattered beams, respectively. Because typical Raman scattering cross-sections are much smaller than those of fluorescent processes, however, Raman spectroscopy is limited in its ability to detect molecules at low concentrations.

**Fig. 1:**
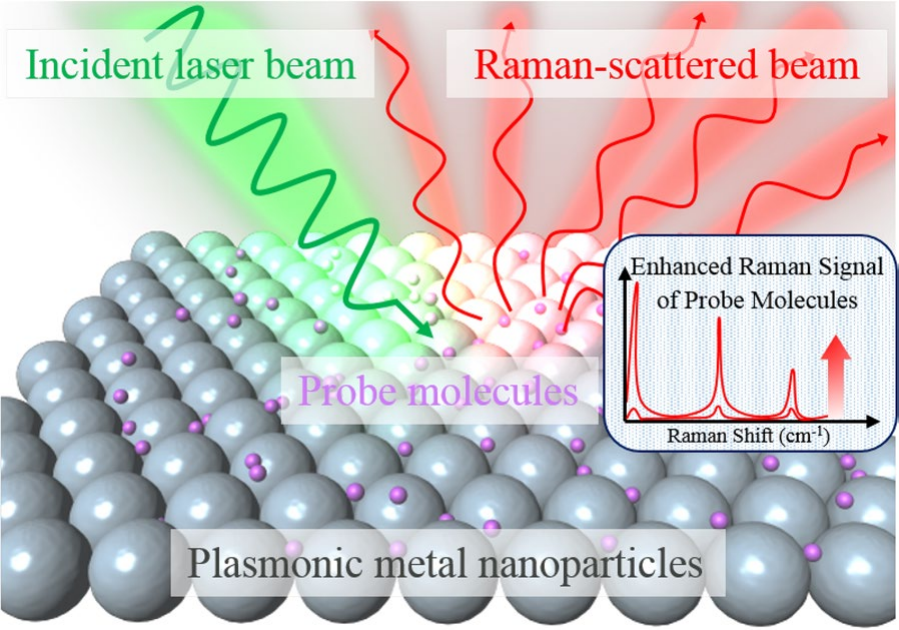
Schematic diagram of the SERS (surface-enhanced Raman scattering) process

The weak intensity of a Raman signal may be enhanced using the localized surface plasmon resonance (LSPR) generated in the near-field of metallic nanostructures [4–7]. Molecules adjacent to the metallic nanostructures exhibit significantly enhanced Raman signals [8–11]. A schematic diagram of the SERS process is presented in Fig. 1. The SERS intensity of a molecule may be described as: [12]

2 $${\text{P }} \propto N\cdot \sigma_{SERS} \cdot \frac{{\left| {E_{loc} } \right|^4}}{{\left| {E_{0} } \right|^4}} \cdot \left| {E_{0} } \right|^{2} ,$$

where N is the number of Stokes-active scatterers (or number of molecules within the laser focal volume), *σ*_*SERS*_ is the scattering cross-section (or chemical enhancement factor), and *E*_*loc*_ and *E*_0_ are the amplitudes of the enhanced and incident electric field, respectively. |*E*_0_|^2^ is the incident laser power. Equation (2) indicates that the SERS signal is proportional to the fourth power of the field enhancement factor ($\frac{{\left| {E_{loc} } \right|}}{{\left| {E_{0} } \right|}}$); therefore, a field enhancement, thanks to the resonant excitation by surface plasmons in the metallic nanostructures, is highly desirable for effectively collecting SERS spectra. The sub-wavelength region of a highly localized electromagnetic field is called the “hot-spot” [13–17].

### Localized surface plasmon resonance (LSPR)

**Fig. 2:**
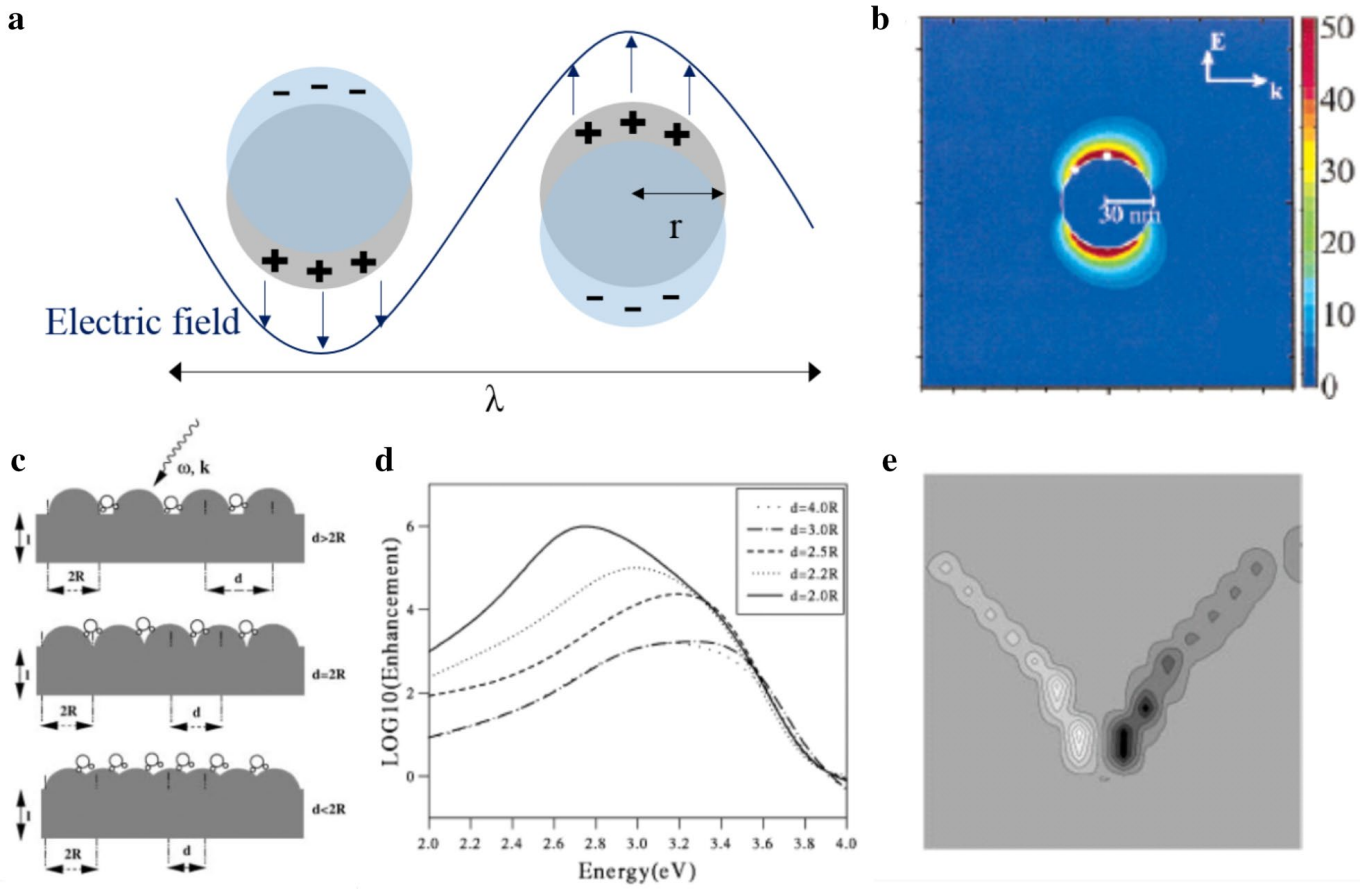
Principle underlying the localized surface plasmon resonance (LSPR) phenomenon and the enhancement in the electric field generated by an LSPR. **a** Electromagnetic field interactions with a metal nanoparticle. **b** Simulation of the electric field distribution near a spherical metal nanoparticle. **c** Optical models of rough metal surfaces prepared with different metal gap sizes (*d* corresponds to the distance between two cylinders, and *R* is the radius of the metal cylinder). **d** Calculated electric field enhancements obtained from different spacings, using the geometry in (**c**). **e** Divergence of the electric field at the sample for 2*R* = *d*, from an incident beam at 2.7 eV. **b** Reproduced with permission from Ref. [22], © 2003, American Chemical Society. **c**–**e** Reproduced with permission from Ref. [23], © 1996, American Physical Society

The LSPR is a non-propagating surface plasmon at the surface of an isolated metallic nanostructure [18–20]. The interaction between an electromagnetic (EM) wave and a curved metallic surface applies a restoring force to oscillating electrons and amplifies the EM field along the surface of the metallic nanostructures due to the resonance [21], as shown in Fig. 2a. The EM field enhancement on the surface of a simple spherical metallic nanostructure may be explained using Mie theory. The polarizability *α* is defined according to

3 $$\alpha = 4\pi R^{3} \frac{{\varepsilon - \varepsilon_{m} }}{{\varepsilon + 2\varepsilon_{m} }},$$

where R is the radius of the metal nanoparticle, *ɛ* is the permittivity of the metal, and *ɛ*_*m*_ is the permittivity of the surrounding medium. As the value of *ɛ* approaches −2*ɛ*_*m*_ (the resonance frequency), the value of the polarizability reaches its maximum. As the polarization increases, the electric field in the vicinity (near-field) of the metallic nanostructure is enhanced. The localization of the electric field near the surface of the metal nanoparticle may be modeled using finite-difference time-domain (FDTD) calculations, as shown in Fig. 2b [22]. The electric field enhancement effect may be increased using arrays of metallic nanoparticles separated by metallic nanogaps. The relationship between the electric field enhancement and the metallic nanogap size may be calculated using a semicylinder array model, as shown in Fig. 2c, d [23]. If the distance between two metallic semicylinders (*d*) corresponds to twice the radius of a semicylinder (*d* = 2*R*), the electric field enhancement reaches its maximum. A 10^6^ enhancement value is obtained from conditions in which *d* = 2R, corresponding to 10^3^ times the value obtained for *d* = 4*R*. The electric field divergence near the metal nanogap for *d* = 2R at a resonance frequency of 2.7 eV is illustrated in Fig. 2e. The electric field was highly enhanced in the metallic nanogap region [24–26].

## Metal nanoparticle assembly and bottom-up lithography

### Shape control over metal nanoparticles

**Fig. 3:**
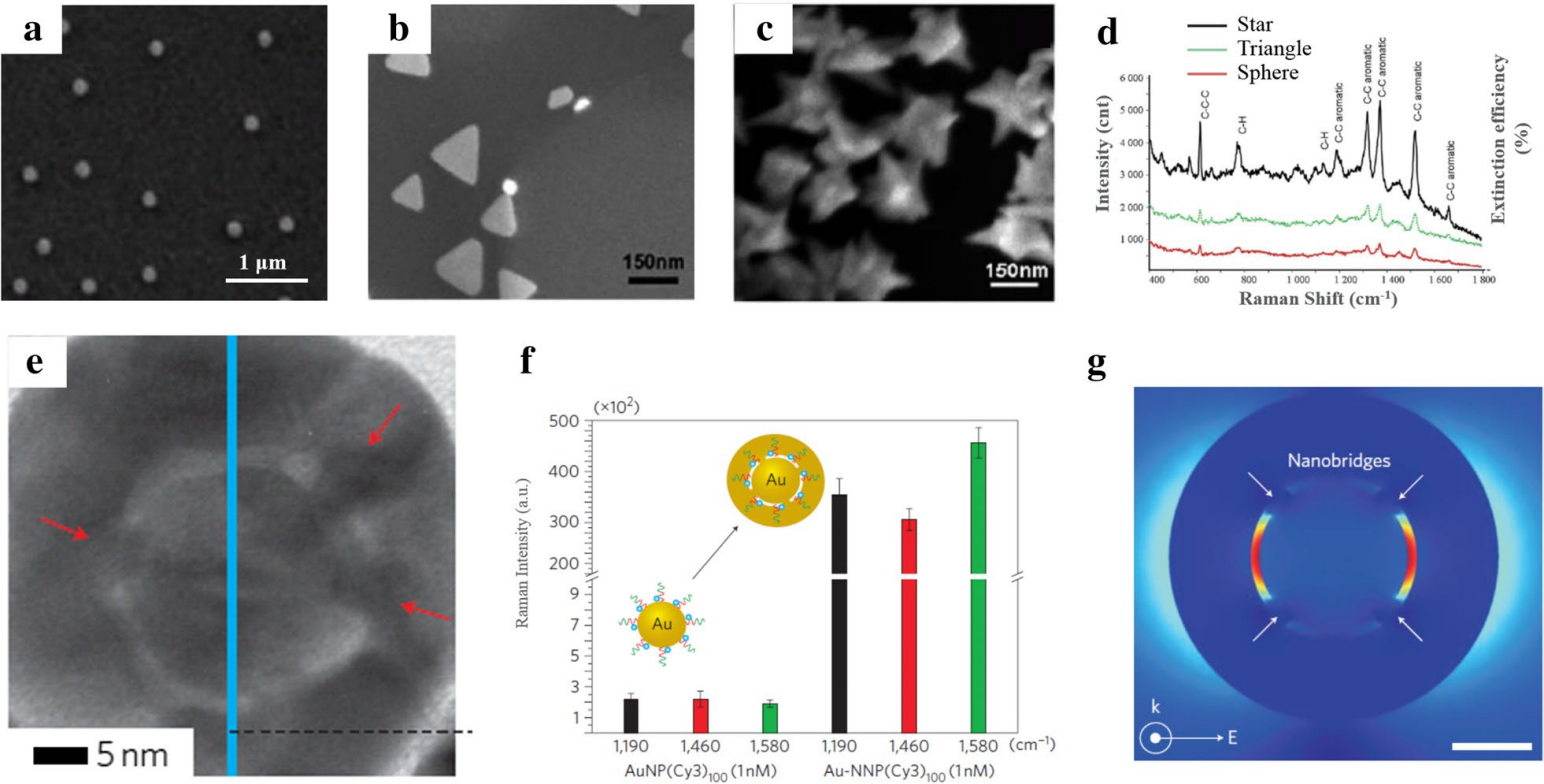
Synthesis of metal nanoparticles. Scanning electron microscopy (SEM) images of **a** gold nanospheres, **b** nanotriangles and **c** nanostars. **d** SERS spectra of R6G molecules measured from three different nanoparticles. **e** HRTEM image of a nanobridged nanogap particle. **f** Comparison of the SERS signal intensity produced in the presence or absence of a nanogap inside of a nanoparticle. **g** Calculated electric field intensity distributions at the metallic inner nanogap. **a**–**c** Reproduced with permission from Ref. [30], © 2014, The Royal Society of Chemistry. **e**–**g** Reproduced with permission from Ref. [32], © 2011, Nature Publishing Group

Controlling the shapes of metal nanoparticles offers a simple strategy for boosting the electric field enhancement factor near a metallic nanostructure [27–29]. As the shape of a spherical metal nanoparticle changes to triangular or star shapes, as shown in Fig. 3a–c, the electric field becomes intensified at the sharp edges of the metal nanoparticle [30]. This effect provides a high-intensity SERS signal, as shown in Fig. 3d. The SERS intensity obtained from star-shaped nanoparticles is much higher than that obtained from triangle-shape particles or metal nanospheres. The electric field enhancement effects at a metallic tip are attributed to the lightning rod effect [31]. The ratio of the surface area to the volume of the corresponding structures is a good indicator of the electron density, which is proportional to the electrostatic field. Therefore, as the edge of a metallic nanoparticle gets sharper, the electric field enhancement effect increases. Instead of creating sharp metal nanoparticles, nanogaps within metal nanostructures may be formed as a good strategy for generating a highly enhanced electric field [32]. During the formation of metal nanoparticles, a hollow gap 1 nm in size between metal nanoparticles may be produced by adopting DNA-assisted metal deposition techniques, as shown in the high-resolution transmission electron microscopy (HRTEM) image presented in Fig. 3e. The SERS intensity increases by two orders of magnitude compared to metal nanoparticles without hollow gaps, as shown in Fig. 3f, due to plasmonic coupling between the metal core and the metal shell (Fig. 3g).

### Direct self-assembly of metal nanoparticles

**Fig. 4:**
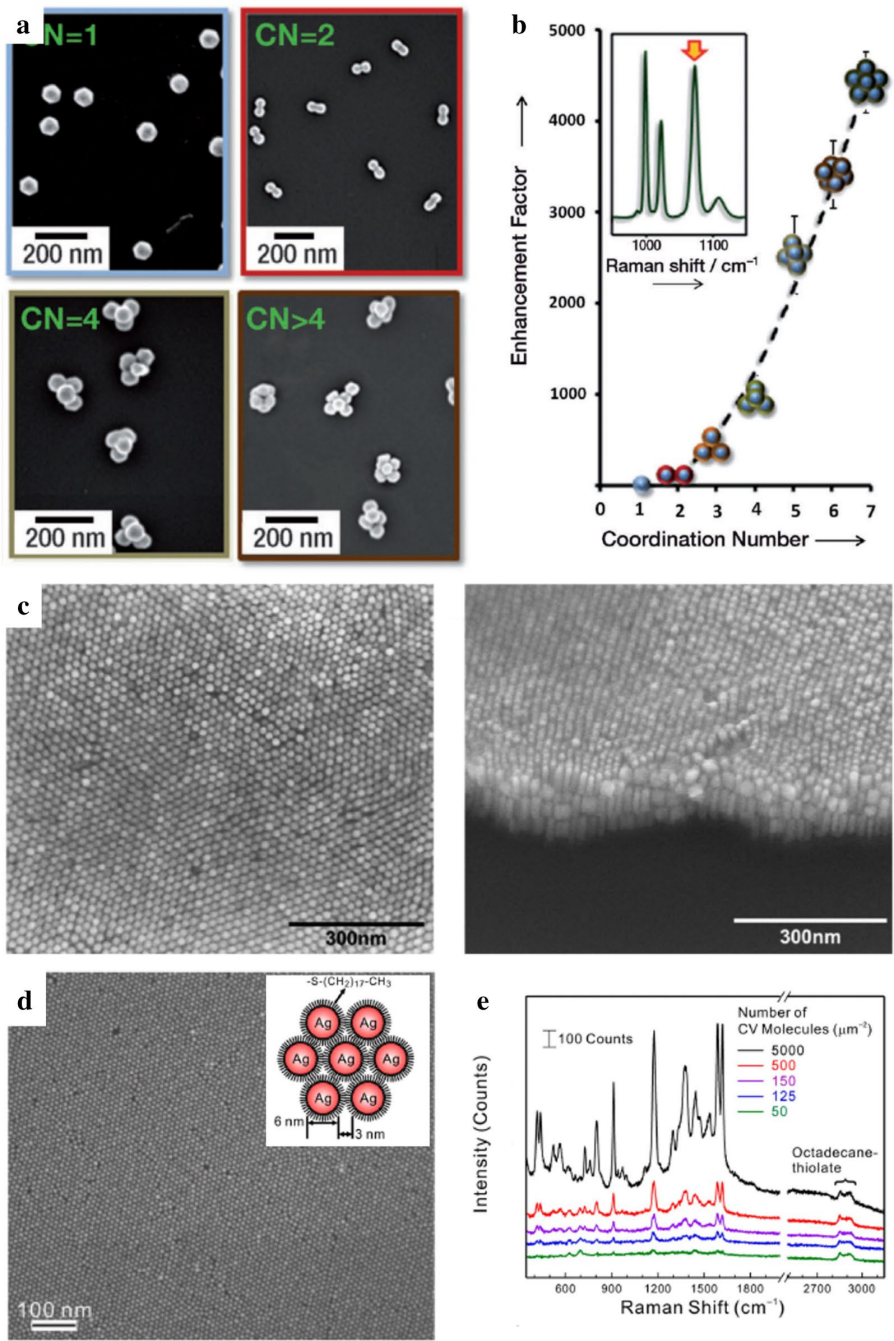
Self-assembly of plasmonic metal nanoparticles. **a** SEM images of gold nanoclusters prepared with different coordination numbers. **b** SERS enhancement factors for different coordination numbers of gold nanoclusters. **c** High-resolution SEM images of self-assembled gold nanorod (GNR) arrays. **d** SEM image of a silver nanoparticle monolayer. **e** SERS spectra of crystal violet molecules, obtained from the sample shown in (**d**). **a**, **b** Reproduced with permission from Ref. [35], © 2012, John Wiley and Sons. **c** Reproduced with permission from Ref. [36], © 2012, John Wiley and Sons. **d**, **e** Reproduced with permission from Ref. [37], © 2015, American Chemical Society

The self-assembly of metal nanoparticles presents one of the simplest methods of producing highly dense intermetallic nanogaps [33, 34]. Solvent evaporation applies an inter-particle capillary force that promotes metallic nanoparticle packing. Colloidal emulsions formed by an ultrasonic homogenizer produce assembled metal nanoparticle clusters with a variety of coordination numbers as the solvent evaporates [35]. A density gradient centrifugation process may be used to sort the metal nanoparticle clusters, as illustrated in Fig. 4a. The electric field enhancement effect near the metal nanoparticle cluster has been shown to increase as the coordination number increases (Fig. 4b). These results demonstrated that the self-assembly of metal nanoparticles can provide highly efficient hot-spots through plasmonic coupling among adjacent metal nanoparticles.

The convective assembly of huge numbers of metal nanoparticles has been used to produce highly dense and sensitive metallic hot spots, as shown in Fig. 4c [36]. The initiated nucleation of gold nanorod (GNR) arrays can lead to the growth of ultra-high density arrays during solvent evaporation. A hexagonal close-packed (HCP) ordering of GNRs is formed by precisely controlling the solution concentration and temperature. Well-aligned metal nanoparticles provide uniform SERS signals.

The assembly of metal nanoparticles using Langmuir–Blodgett troughs has been used to generate highly close-packed metal nanoparticle monolayers. Alkanethiolate ligand-treated silver nanoparticles and certain analytes can be used to control the distance between close-packed silver nanoparticles in a monolayer to provide uniform and planar EM field distributions [37]. An SEM image of a monolayer of ligand-treated silver nanoparticles is shown in Fig. 4d. This highly uniform array of silver nanoparticles illustrates the linear relationship between the SERS intensity and the concentration of analyte. The detection of 50 crystal violet molecules per μm^2^ is possible using this type of array (Fig. 4e).

### Colloidal lithography

**Fig. 5:**
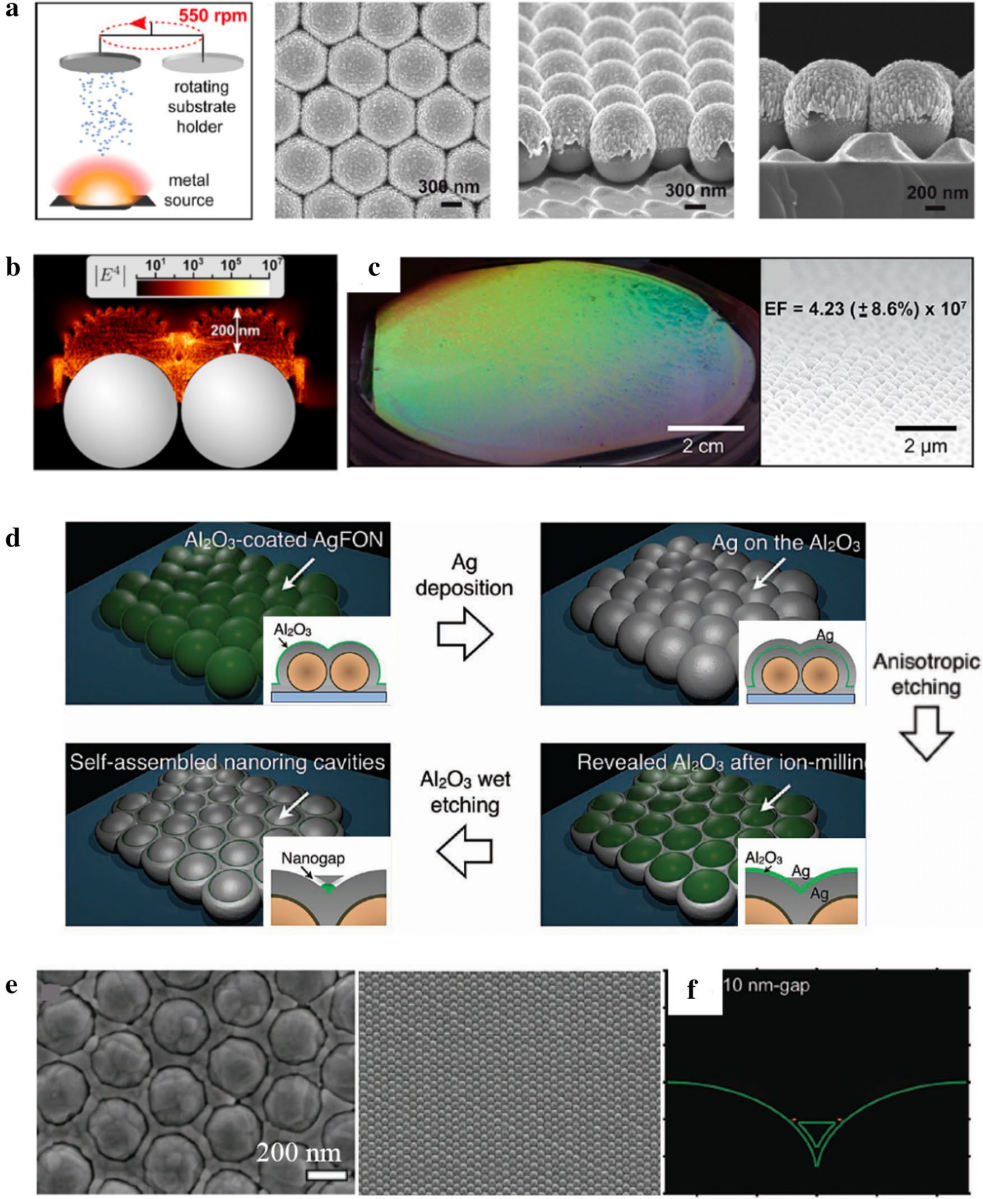
Hot-spot generation using a colloidal crystal template. **a** Schematic diagram of the metal deposition method applied to a rotating substrate, and SEM images of the AgFON with a high surface roughness. **b** Calculated electric field intensity distributions near the AgFON substrate coated with a 200 nm silver film. **c** Uniformity of the nanopatterns at the wafer scale, and tilted SEM image of the AgFON film, showing its high degree of uniformity. **d** Schematic diagram of the procedure used to fabricate 10 nm metallic nanogaps from nanoring cavities. **e** SEM image of a FON substrate prepared with 10 nm nanogaps. **f** Calculated electric field intensity distributions near the FONs prepared with 10 nm nanogaps. **a**–**c** Reproduced with permission from Ref. [39], © 2013, American Chemical Society. **d**–**f** Reproduced with permission from Ref. [40], © 2013, John Wiley and Sons

Colloidal lithography is a versatile method for fabricating high-density plasmonic nanogap arrays [38]. The deposition of a silver film on the surface of a colloidal monolayer can produce rough metallic nanostructures on the colloidal particle array (Fig. 5a) [39]. An Ag film over a nanosphere (AgFON) platform has been generated by rotating the substrate during the metal deposition process. FDTD simulations have revealed that the electric field enhancement is maximized at the point at which adjacent colloidal particles meet, as shown in Fig. 5b. Figure 5c shows that the spin-cast deposition of colloidal metal nanoparticles onto silicon substrates generates uniform hexagonally close-packed arrays.

Other approaches to LSPR generation include deposition of a dielectric interlayer onto the top surface of an AgFON using the atomic layer deposition method [40]. As shown in the experimental procedure used to prepare metallic nanogaps on AgFON substrates (Fig. 5d), an aluminum oxide (Al_2_O_3_) layer as well as the subsequently deposited Ag film produced a metal/dielectric/metal multilayer that is useful for generating plasmonic coupling at the metallic nanogaps after partially removing the aluminum oxide layer using a buffered oxide etchant. Figure 5e shows scanning electron microscopy (SEM) images of a nanoring cavity on an AgFON substrate. FDTD simulation data presented in Fig. 5f reveal that the intensity of the electromagnetic field is highly localized at the 10 nm gaps of the silver nanostructures. This approach provided a maximum enhancement factor of 10^8^, higher than that obtained from conventional AgFON SERS templates, typically 10^7^.

### Block copolymer lithography

**Fig. 6:**
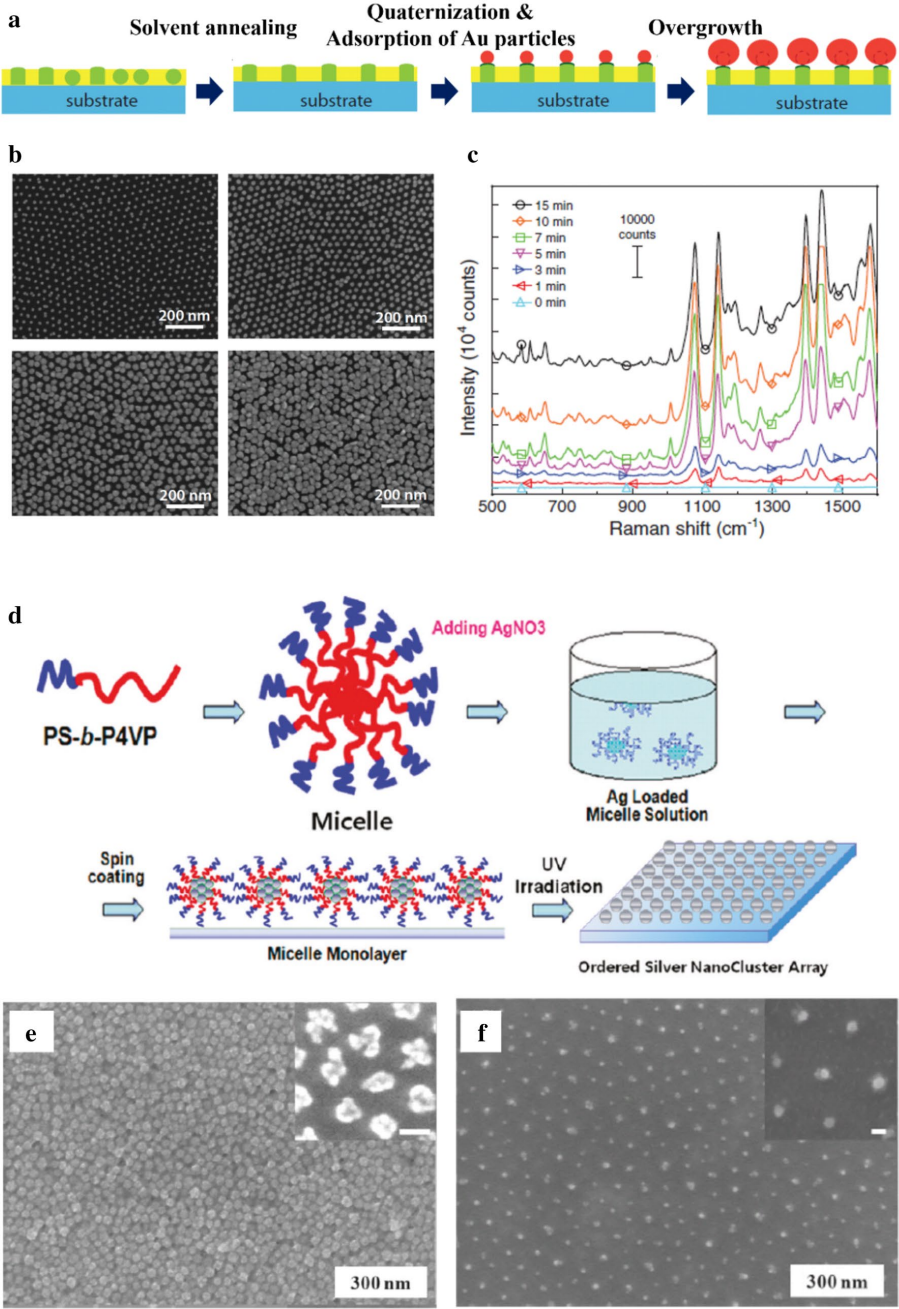
Hot-spot generation using block copolymer lithography. **a** Schematic diagrams illustrating the procedures used to fabricate a block copolymer-assisted SERS substrate. **b** SEM images of gold nanoparticle arrays prepared with different overgrowth times of 1, 3, 7, or 15 min. **c** SERS spectra of 4-aminobenzenethiol, obtained from gold nanoparticles with different overgrowth times. **d** Schematic diagrams illustrating the block copolymer micelle-assisted silver nanocluster assembly. **e**, **f** SEM image of silver nanocluster arrays obtained by controlling the weight percent of PS and the PS molecular weight: **e** 10,400 g/mol or **f** 122,000 g/mol, using similar weight percentages of P4VP. **a**–**c** Reproduced with permission from Ref. [44], © 2011, John Wiley and Sons. **d**–**f** Reproduced with permission from Ref. [45], © 2012, American Chemical Society

The self-assembly of block copolymers provides ordered polymeric nanostructures with a variety of geometries [41–43]. The selective deposition of metals onto one part of a polymer produces well-ordered metal nanostructures. The procedure used to fabricate gold nanosphere arrays using block copolymer self-assembly is illustrated in Fig. 6a [44]. A thin film of the self-assembled block copolymer PS-b-P4VP (polystyrene-b-poly(4-vinylpyridine)) produces hexagonally ordered P4VP cylinders with a PS surrounding medium due to the segregation of the hydrophilic and hydrophobic parts. Gold nanoparticles slightly larger than the P4VP domains were attached to the P4VP sites through electrostatic interactions. The gap size between adjacent gold nanoparticles could be tuned by controlling the metal overgrowth. Figure 6b shows SEM images of hexagonally arranged gold nanoparticle arrays with different overgrowth times. As the overgrowth time increased from 0 min to 3, 7, and 15 min, the average size of the metal nanoparticles increased from 16.6 nm to 24.8, 32.7, and 36.5 nm. Correspondingly, the gap between the particles decreased from 23 nm to 14.6, 9.5, and 4.1 nm, respectively. Figure 6c shows SERS spectra of 4-aminobenzenethiol in the presence of substrates prepared using different overgrowth times. The SERS intensity measured from the 7 min overgrowth substrate was the highest because the wavelength of the LSPR peak matched the wavelength of the incident light and the Stokes Raman shift of 4-aminobenzenethiol. As an alternative to attaching the gold nanoparticles onto the block copolymer surfaces, the metal precursor has also been reduced onto block copolymer micelles to form compartments and assemble the metal nanoparticles within the micelle. As shown in Fig. 6d [45], silver nanoparticles could be loaded into the P4VP regions of a PS-b-P4VP block polymer micelle by adding AgNO_3_ and NaBH_4_. Silver nanoparticle clusters could be packed and remained on the substrate in a hexagonal arrangement after monolayer deposition of the micelle and the subsequent removal of the PS-b-P4VP block polymer. The distance between neighboring silver clusters was determined by the molecular weight percent of PS, whereas the diameter of the silver nanocluster was preserved. Figure 6e, f show silver nanocluster arrays with different inter-particle distances.

## Top-down nanolithography

### Electron beam lithography

**Fig. 7:**
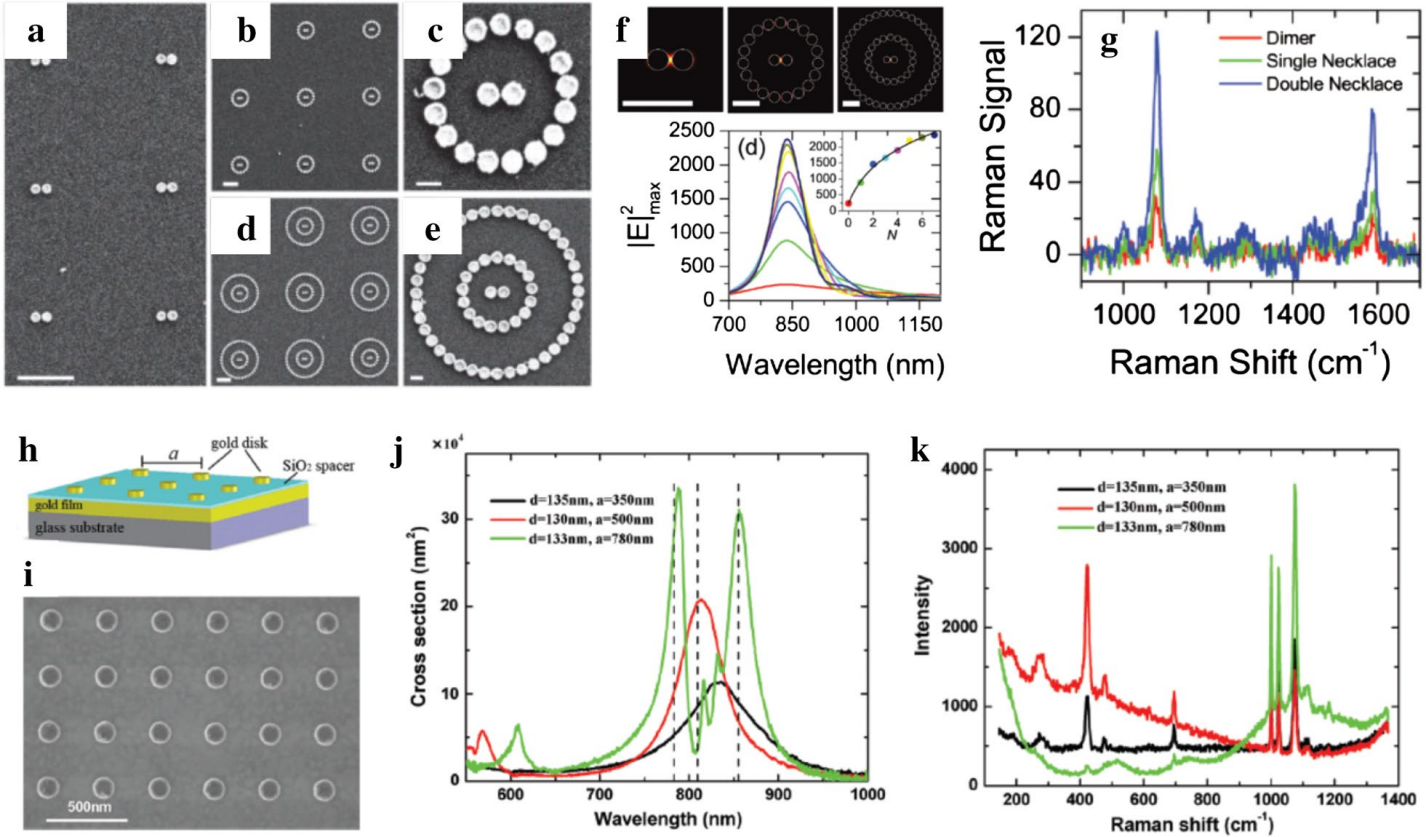
Electron beam lithography SEM images of **a** a gold dimer, **b**, **c** a dimer/heptadecagon array, and **d**, **e** a dimer/heptadecagon/tetracontagon array. **f** Calculated electric field intensity distributions at the isolated dimer, dimer/enneadecagon, and dimer/enneadecagon/tetracontagon. Comparison of the electric field intensity obtained from different numbers of necklaces. **g** Comparison of the SERS spectra obtained from an isolated dimer, a dimer/enneadecagon, and a dimer/enneadecagon/tetracontagon. **h** Schematic diagram of gold nanodisk arrays prepared on a silica-coated gold film to form double resonance systems. **i** SEM image of gold nanodisk arrays prepared on a silica film-coated gold film. **j** Extinction spectra obtained from three different gold nanodisk arrays with different diameters and periods. **k** SERS spectra of benzenethiol obtained from three different geometries. **a**–**g** Reproduced with permission from Ref. [50], © 2012, American Chemical Society. **h**–**k** Reproduced with permission from Ref. [51], © 2010, American Chemical Society

Electron beam (e-beam) lithography can be used to fabricate desired high-resolution nanostructures that are not easily prepared using other fabrication methods [46, 47]. The high degree of freedom available for e-beam lithography has enabled studies of the basic principles underlying electric field enhancements by well-designed metallic nanostructures [48, 49]. Metal nanostructures with high electric field confinement effects have been prepared using concentric rings of plasmonic necklaces deposited near metal nanoparticles dimers, as shown in Fig. 7a–e [50]. The introduction of concentric rings around metal nanodisk dimers significantly concentrates the electric field at the gap within the dimer region. The intensity of the electric field increases as the number of necklaces increases, as shown in the FDTD simulations presented in Fig. 7f. The SERS signal of a *p*-mercaptoaniline (pMA) monolayer on the surface of a double necklace was much higher than that obtained from a single necklace or a single dimer structure (Fig. 7g). The LSPR wavelength can also be tuned via precise control over metal nanostructures. The fabrication of a gold nanodisk on the top surface of a silica spacer-coated gold film creates a double-resonance plasmonic substrate, as shown in Fig. 7h, i [51]. The double resonance between the localized surface plasmons (LSPs) and the surface plasmon polaritons (SPPs) occurs due to variations in the distance between the gold nanodisks. The distance between gold disks is the main factor that determines the position of the plasmon resonance. As shown in Fig. 7j, a distance between two adjacent gold nanodisks of 780 nm produces dominant LSPs and SPPs, and the extinction spectra exhibit two main peaks (the peak position at the higher wavelength is the LSP). The tunability of the LSPR position is beneficial for SERS applications because the intensity of the SERS signal is higher if the LSPR wavelength is located between the wavelength of excitation and the Raman signal. For example, nanodisk arrays separated by a distance of 350 or 500 nm distance displayed a higher SERS intensity than nanodisk arrays separated by a distance of 750 nm in the presence of benzenethiol excited at 421 cm^−1^, because the LSPR position of the nanodisk arrays with 350 and 500 nm separation distances is much closer to the wavelength of excitation (783 nm) and Raman signal than is the case for the arrays with a 750 nm separation distance (Fig. 7k).

### Nanoimprint lithography

**Fig. 8:**
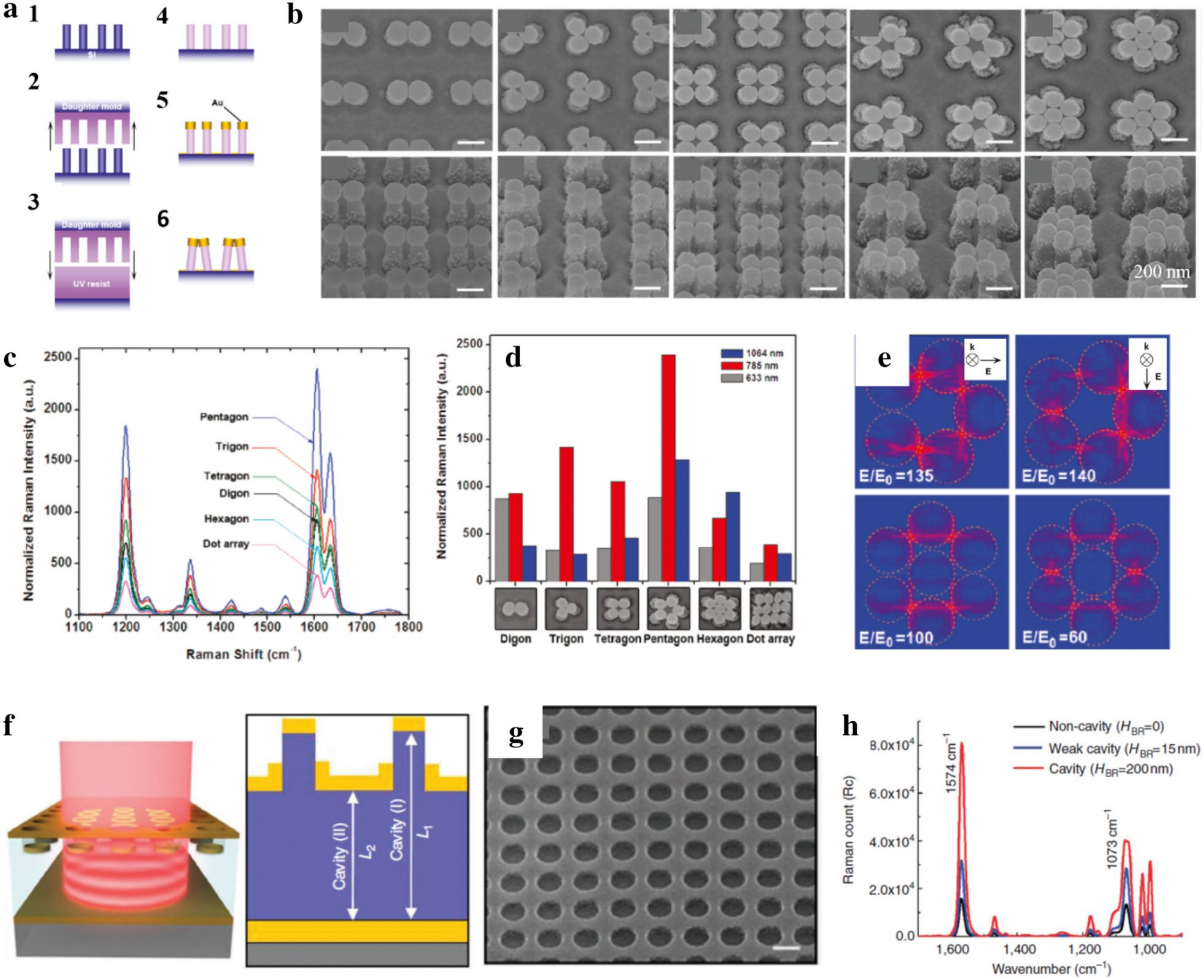
Nanoimprint lithography **a** Procedure used to fabricate gold nanofinger patterns. **b** SEM images of the assembled nanofinger arrays for use in preparing digon, trigon, tetragon, pentagon, or hexagon configurations. The *first row* shows a top-view and the *second row* shows a tilted view. **c** Raman spectra of BPEs obtained from six different geometries. **d** Comparison of the SERS signals obtained from six different geometries. **e** FDTD simulations of the electric field intensity distributions measured near the pentagon and hexagon structures. **f** Double resonance plasmonic structures fabricated from nanoimprint lithography. **g** SEM image of a *top view* of the plasmonic structures. **h** SERS spectra of three different cavity *shapes*. **a**–**e** Reproduced with permission from Ref. [56], © 2011, American Chemical Society. **f**–**h** Reproduced with permission from Ref. [57], © 2011, Nature Publishing Group

Nanoimprint lithography is useful for the generation of highly reproducible nanostructures [52–55]. The limitations of nanoimprint lithography in SERS applications have been the high fabrication costs associated with the master mold and the limited resolution at which few nm gap structures may be generated. To overcome these problems, several approaches to creating highly efficient metallic nanogaps with effective electric field confinement have been developed. The coalescence of nanocylinder arrays using capillary force is useful for fabricating nanogaps that enhance the electric field confinement [56]. After depositing gold onto the heads of a polymeric nanocylinder array, introduction of an analyte solution dropped onto the arrays induced the nanocylinder arrays to close under capillary forces. Figure 8a, b show the fabrication steps and SEM images of the resulting assembled nanocylinder arrays. The SERS signals obtained from trans-1,2-bis (4-pyridyl)-ethylene (BPE) in different geometries are compared in Fig. 8c, d. Symmetric structures, such as diagonal, tetragonal, and hexagonal arrays, exhibited relatively low intensities compared to the asymmetric trigonal and pentagonal structures, as supported by the FDTD calculations (Fig. 8e).

Cavity coupling among plasmonic nanostructures can be obtained using molding techniques and subsequent metal deposition, as shown in the schematic diagrams of Fig. 8f. [57]. Figure 8g shows the square array of cavities. Plasmonic coupling effects occur in both the L_1_ and L_2_ cavities to provide a tunable plasmonic resonance wavelength by controlling the cavity size. A well-designed cavity may produce a highly localized electric field near the metal surface, resulting in a high-intensity SERS signal from benzenethiol, in contrast to the non-efficient cavity, as shown in Fig. 8h. Interestingly, the band at 1574 cm^−1^ was higher than the 1073 cm^−1^ peak band because the 1574 cm^−1^ band is close to the approximate maximum SERS intensity range of $\left( {\lambda_{\text{LSPR}} \approx {{\left( {\lambda_{\text{Ex}} + \lambda_{\text{Raman}} } \right)} \mathord{\left/ {\vphantom {{\left( {\lambda_{\text{Ex}} + \lambda_{\text{Raman}} } \right)} 2}} \right. \kern-0pt} 2}} \right)$.

### Laser interference lithography

**Fig. 9:**
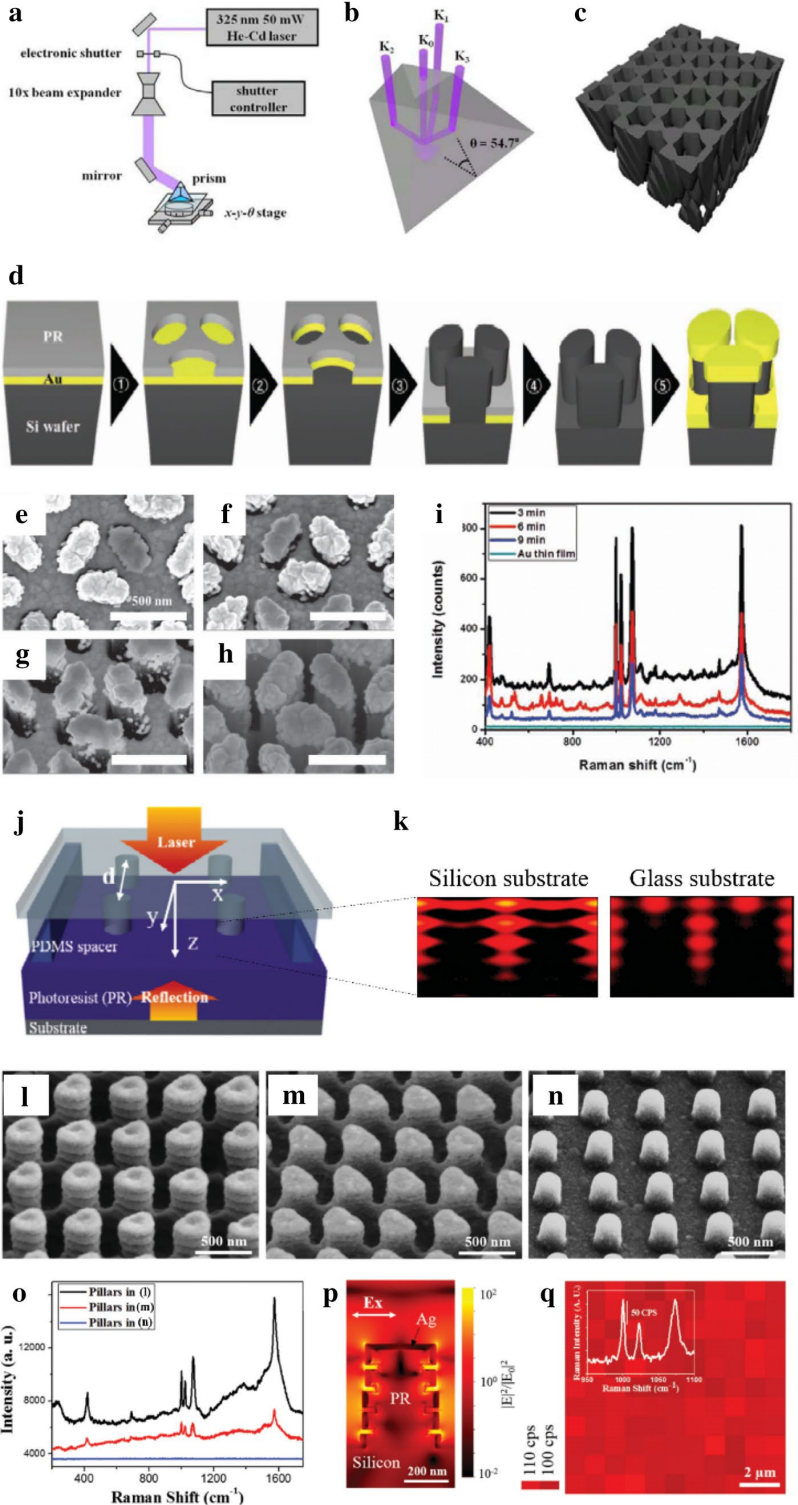
Laser interference lithography. **a** Optical setup used for prism holographic lithography. **b** Geometry of the prism used to obtain the 4-beam interference. **c** 3D interference patterns calculated from the 4-beam interference patterns using the FCC geometry. **d** Procedures used to fabricate a hexagonally ordered Au-capped silicon nanowire using prism holographic lithography. **e**, **f** Au-capped silicon nanowire structures obtained over a 3 min etching time. **g**, **h** Au-capped silicon nanowire structures obtained over 6 min (**g**) or 9 min (**h**) etching times. **i** Comparison of the SERS spectra obtained from Au-capped silicon nanowire arrays prepared with different etching times. **j** Schematic diagram of the phase shift lithography setup. **k** Laser interference patterns applied to the different underlying substrates. A silicon wafer has a larger refractive index than a glass wafer, resulting in a high-intensity reflected beam that creates a vertical standing wave effect. **l**, **m** Nanopillar arrays generated using a silicon substrate (**l**) or a glass substrate (**m**). **n** Nanopillar arrays with smooth side walls. **o** SERS spectra of benzenethiol obtained from three different nanopillar arrays, (**i**–**n**). **p** FDTD simulation of the electric field intensity distributions near the undulating nanopillar array structures (**i**). **q** SERS spectra mapping, collected in 1 µm intervals, to measure the uniformity of the SERS substrate. **a**–**i** Reproduced with permission from Ref. [63], © 2012, John Wiley and Sons. **j**–**q** Reproduced with permission from Ref. [65], © 2015, John Wiley and Sons

Laser interference lithography uses interference among several monochromatic light sources with different wavevectors, polarizations, and phases to fabricate periodic nanostructures [58–60]. The laser interference patterns produce highly uniform and reproducible SERS substrates [61]. Complex alignment of an optical set-up may be avoided by using prism holographic lithography and phase-shift lithography with a diffraction grating. Prism holographic lithography uses a well-designed prism to split incident light into several beams with a certain wavevector profile [62]. The use of a top-cut triangular pyramid prism (Fig. 9a, b) produces a four-beam interference pattern from a single laser exposure, which generates face-centered cubic (FCC) structures, as shown in Fig. 9c. The interference pattern is transferred to the thin film of a photoresist to generate hexagonal arrays of three elliptical holes [63]. The patterned photoresist film is used as an Ar-milling mask to remove exposed areas of gold. The resulting patterned gold structures are used as a catalyst to etch the underlying Si wafer in a solution containing deionized water, hydrogen peroxide (H_2_O_2_), and hydrofluoric acid (HF). Finally, well-ordered silicon nanowires (SiNWs) have been fabricated with various lengths, depending on the etching rate. Highly uniform silicon nanowires can act as hot spots after Au deposition. The fabrication procedures and final nanostructures are illustrated in Fig. 9d, e–h. A small gap between top and bottom gold films provides an efficient electric field enhancement effect, resulting in a high-intensity benzenethiol SERS signal, as shown in Fig. 9i.

Laser interference patterns generated by a diffraction grating are advantageous for the fabrication of reproducible 2D and 3D periodic nanostructures over large areas (comparable to the laser beam size) using a single laser exposure [64]. Unlike prism holographic lithography, phase-shift lithography uses the interference among several diffracted light beams split by a diffraction grating. The interference patterns of the diffracted beams generated in the Fresnel regions are transferred to the photoresist film to generate ordered nanopillar arrays with a rectangular arrangement. During the laser interference patterning process, beams reflected from the interface between the photoresist and the underlying substrate produce a vertical standing wave by combining with the incident light to generate a vertical undulating pattern on the sides of the nanopillar arrays [65]. A schematic diagram of the laser interference lithography method using a diffraction grating is shown in Fig. 9j. FDTD simulation data reveal that substrate with higher refractive index causes higher intensity of the reflected beam, thereby resulting in clearer undulating patterns on the side walls of nanopillars (Fig. 9k). As shown in Fig. 9l, m, nanopillar arrays produced on a silicon wafer display much clearer undulating patterns, whereas nanopillar arrays formed on a glass wafer have indistinct undulating patterns. The smooth-surface nanopillar arrays were prepared and the SERS spectra of benzenethiol were measured on three different substrates (Fig. 9o). The intensity of the SERS signal obtained from the undulating nanopillar array was much higher than that obtained from the indistinctly undulating nanopillar array, and the smooth surfaces of the nanopillars provided no SERS signal at all. The electric field was significantly enhanced at the metallic nanogaps on the side walls of the undulating nanopillars (Fig. 9p). The undulating nanopillar array provides high uniformity of SERS intensity over wide area (Fig. 9q).

## Nanolithography-free random array generation

### Maskless dry-etched silicon template

**Fig. 10:**
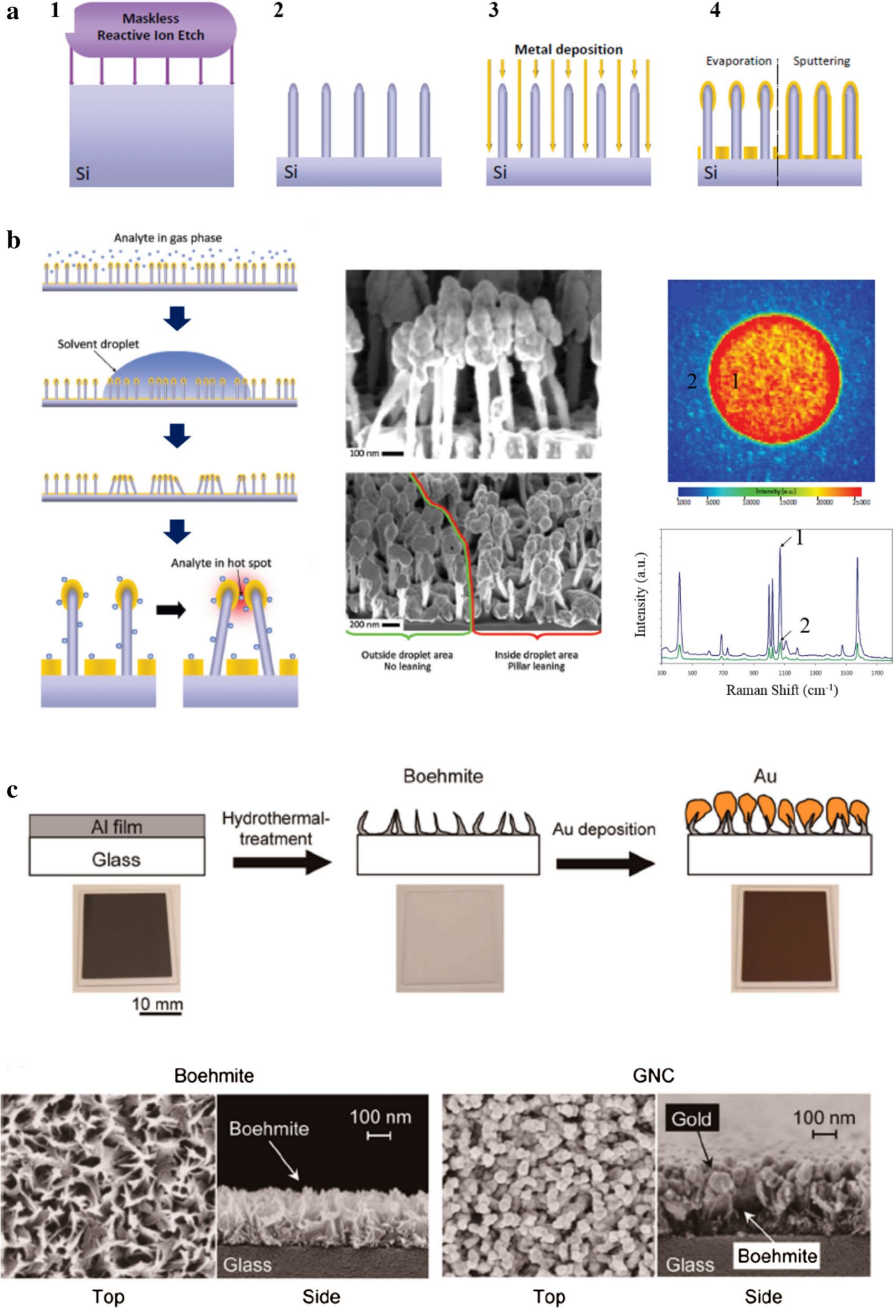
Nanolithography-free random array generation. **a** Procedure used to fabricate metal nanopillar arrays using a maskless reactive ion etching process. **b** Concept underlying the metal nanopillar array leaning process during the evaporation of solvent (*left panel*), SEM images of the nanopillar arrays (*center panel*), and comparison of the Raman maps of benzenethiol between the droplet contact region and the original samples (*right panel*). **c** Procedure used to fabricate gold nanocoral (GNC) structures having under 10 nm metal nanogaps, using the hydrothermal treatment of an aluminum film and SEM images of the *top* and *side views* of boehmite and gold nanocoral substrates. **a**, **b** Reproduced with permission from Ref. [66], © 2012, John Wiley and Sons. **c** Reproduced with permission from Ref. [67], © 2014, American Chemical Society

SERS substrates have been fabricated without nanolithography techniques by applying the maskless reactive ion etching (RIE) of silicon substrates, as shown in Fig. 10a [66]. Careful control over the RIE process conditions can yield free-standing Si nanopillars with the desired pillar height and width. Silver is deposited onto the random nanopillar arrays by evaporation or sputtering methods. It should be noted that nanopillar leaning is crucial for obtaining large SERS signals, as in the case of the polymeric pillar substrates fabricated by nanoimprint technology. Evaporation deposition was found to be favorable for preparing silicon lumps at the tops of Si pillars during deposition. By contrast, conformal silver coatings formed using sputtering methods resulted in rigid pillars and yielded poor SERS performances. The leaning effect produced metallic nanogaps (hot-spots) during solvent evaporation, and the SERS characteristics of these substrates improved, as shown in Fig. 10b. The droplet contact region produces high-intensity of SERS signals compared to other regions due to the strong plasmonic coupling among the leaning nanopillar arrays. The advantages of maskless patterning without a lithographic step enables the preparation of cost-effective consumable SERS substrates. A SERS enhancement factor on the order of 2.4 × 10^6^ was claimed, and the signal uniformity over large areas, that is, the 4 in. wafer scale, was demonstrated.

### Hydrothermally roughened metal templates

Boiling aluminum thin films coated onto glass transforms a flat metallic films to a sharp-edged leaf-like superstructure of boehmite (Fig. 10c) [67]. Random boehmite arrays have been used as templates for gold deposition to produce gold nanocoral (GNC) surfaces. By optimizing the Au deposition, effective plasmonic nanostructures could be obtained with a gap spacing of <10 nm. During the gold deposition step, a gold film is not generated in the valleys of the boehmite structures, and the growth of gold is initiated on the ledges, resulting in the formation of hot-spots. The analytical enhancement factors of these substrates are on the order of (1–3) × 10^4^, and uniform SERS signals are obtained over large areas. These substrates perform sufficiently well to support their use in the construction of two-dimensional Raman maps for tissue imaging.

### Hybrid methods of random hot-spot generation

**Fig. 11:**
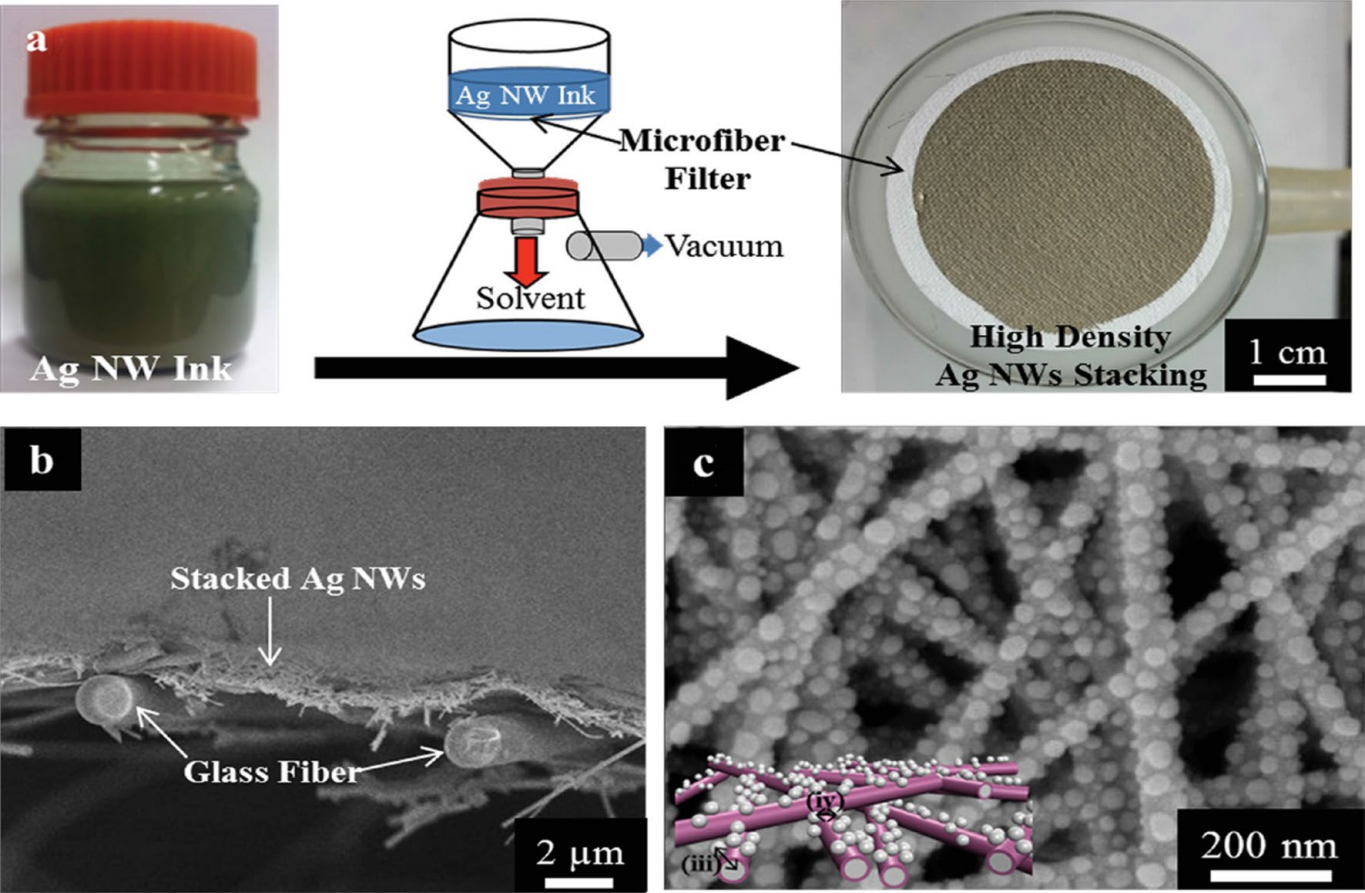
Random hot-spot generation via Ag nanowire stacking. **a** Filtration of an Ag NW solution to produce randomly stacked Ag NWs. **b** SEM image of 3D stacked Ag NWs on a glass filter. **c** SEM image of 3D hybrid nanostructures (Ag nanoparticles/alumina/Ag NWs). **a**, **c** Reproduced with permission from Ref. [68], © 2015, John Wiley and Sons

A hybrid fabrication method involving random stacking of Ag nanowires and vacuum-deposition of Ag nanoparticles, has been reported [68]. The simple filtration of Ag NWs using a glass fiber membrane renders a random stacking of high-aspect-ratio nanowires (Fig. 11a, b). Despite the random configuration, the stacked Ag NWs plates show effective plasmonic characteristics due to the huge number of hot-spots present at the crossing points of the NWs. Plasmonic coupling may be further promoted by decorating the stacked NWs with Ag nanoparticles. The presence of a 10 nm thick alumina interlayer serves as an effective dielectric gap between the NPs and NWs, and the nonwetting properties of the Ag film on the surface facilitates the island-type growth of Ag during vacuum deposition (Fig. 11c). The 3D hybrid plasmonic nanomaterials exhibited near-perfect absorption across the visible wavelength range (averaged reflectance <2 %) and ultrasensitive SERS performances.

SERS substrates consisting of silver nanoislands have been prepared on other supporting plates, such as glass nanopillar arrays or papers [69]. The glass nanopillar arrays were fabricated by reactive ion etching through an annealed Ag nanoisland mask. Thermal evaporation of Ag onto the nanopillar arrays yields a high density of Ag NPs on both the tops and sidewalls of the nanopillars, similar to the nanoislands growth described above in the context of the Ag NW stacked plates. Laser illumination of the Ag films deposited onto paper fibrils can facilitate the formation of highly dense Ag nanoparticles [70]. The quasi-3D distribution of Ag NPs on the paper provides effective SERS signals throughout the detection area.

## SERS-based sensor applications

**Fig. 12:**
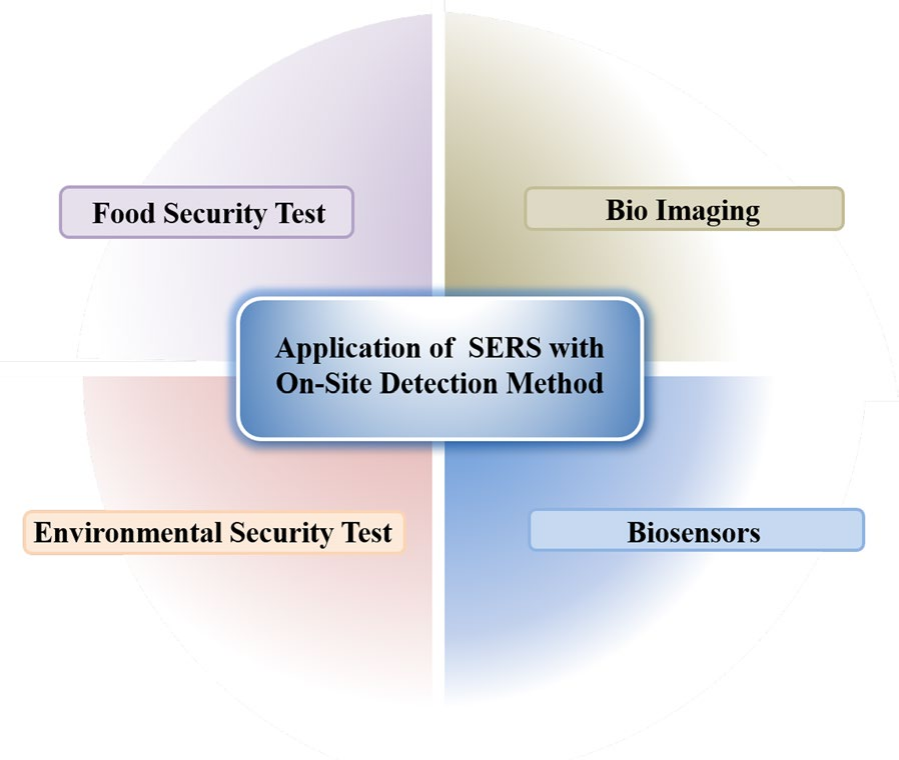
Applications of portable SERS spectroscopy instruments

The simple, sensitive, and rapid detection of trace levels of highly toxic molecules and biomarkers are in urgent demand for environmental monitoring and public health applications [71, 72]. The fabrication of reliable, reproducible, and low-cost SERS substrates are a prerequisite for these general chemical and biosensing applications [73–80]. The development of portable Raman spectrometers can enable a variety of field tools, such as food or environmental security tests, bio-imaging devices, and bio-sensors, as shown in Fig. 12.

### Rapid and on-site detection of analytes using portal Raman systems

**Fig. 13:**
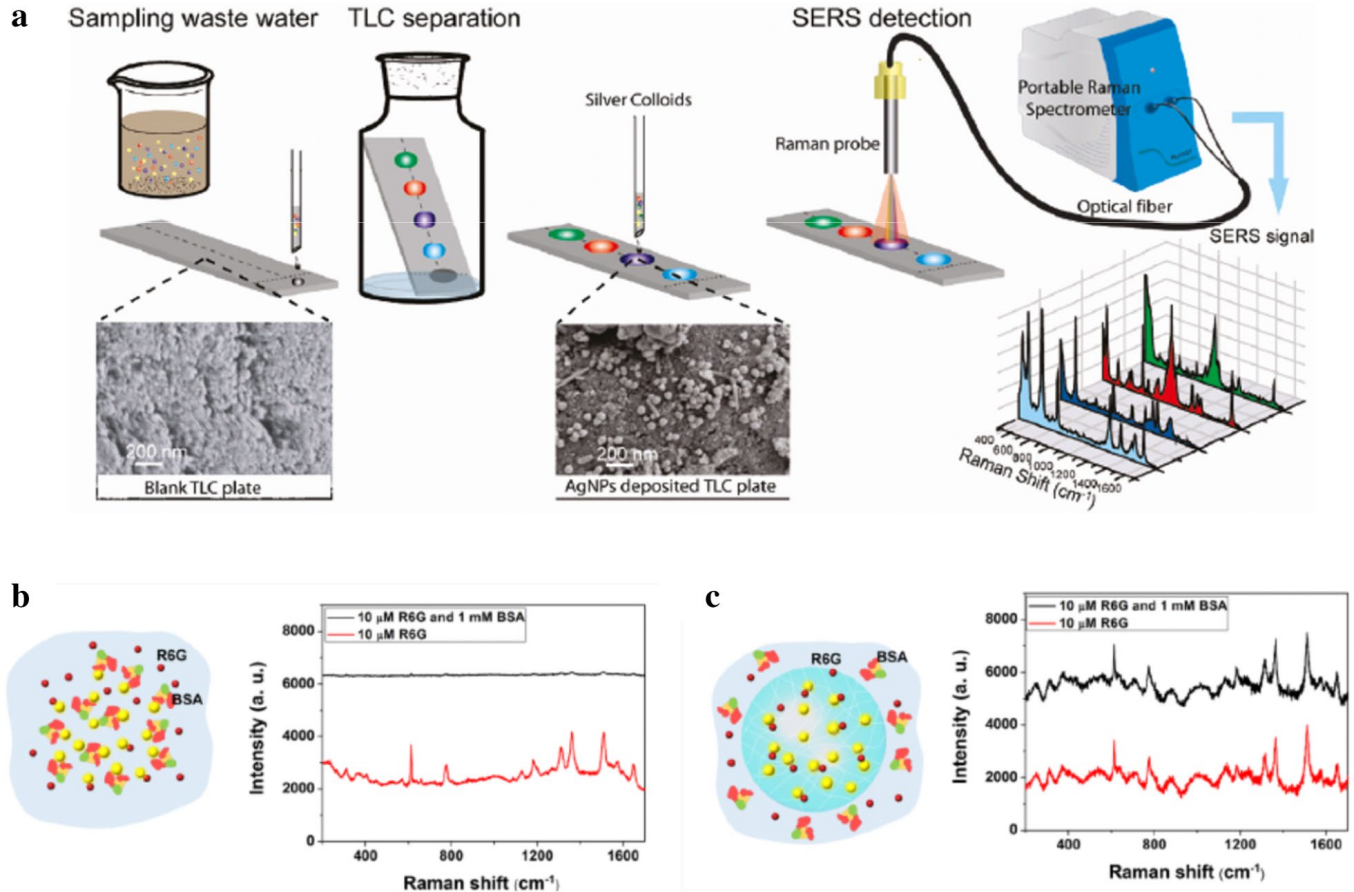
Rapid and on-site detection using portal Raman spectroscopy. **a** Schematic diagram illustrating TLC separation and analyte measurement using a portable SERS substrate. **b**, **c** Detection of R6G from gold nanoparticles mixed with R6G and BSA without a hydrogel (**b**) or with a hydrogel (**c**). **a** Reproduced with permission from Ref. [81], © 2011, American Chemical Society. **b**, **c** Reproduced with permission from Ref. [82], © 2016, American Chemical Society

Despite the high sensitivity of SERS substrates, the need to pre-treat samples limits the practical utility of SERS substrates as molecular sensors. In real environmental systems, analytes are mixed with other molecules, and the separation of target molecules should be conducted prior to collecting Raman measurements. Thin layer chromatography (TLC)-based techniques show promise as a simple process for separating molecules [81]. The TLC approach separates molecules according to the rate at which they travel through a thin layer due to differences in the attractive forces between the molecules and the film, as well as the differential solubility of the molecules in the solvent. Dropping silver particles onto the molecules separated on a TLC plate permits the on-site detection of analytes using a portable Raman spectrometer, as shown in Fig. 13a.

The size-selective separation of analytes is useful for detecting small molecules dispersed in a mixture, such as human blood or commercial beverages. Hydrogels can separate target molecules from a mixture using their small mesh size [82]. Molecules with a size smaller than the mesh size of the hydrogel can diffuse into a gold nanoparticle-encapsulated hydrogel, whereas larger materials are excluded. Figure 13b, c show the effects of a hydrogel on the size-selective diffusion of molecules. Gold nanoparticles that simultaneously come in contact with both rhodamine 6G (R6G) and bovine serum albumin (BSA) do not provide a detectable R6G Raman signal due to interference by BSA; however, encapsulating gold nanoparticles in a hydrogel allows only the R6G molecules to diffuse into the gel, and R6G detection is possible.

### Food and environmental security control

Safety tests conducted using SERS substrate offer a promising method due to the high sensitivity and selectivity of the SERS substrate. Small quantities of toxic molecules on the surfaces of food may be detected using well-designed efficient SERS substrates [83]. Plasmonic nanopillar leaning during solvent evaporation provides a high-intensity SERS signal due to plasmonic coupling among adjacent plasmonic nanopillar arrays. Different concentrations of thiabendazole (TBZ) molecules were introduced onto the surfaces of apples, which were then rinsed with DI water. The rinse water was then introduced to the prepared SERS substrates. The SERS measurements could detect a 7 ppb concentration of TBZ molecules, lower than the Environmental Protection Agency (EPA)’s detection limit.

**Fig. 14:**
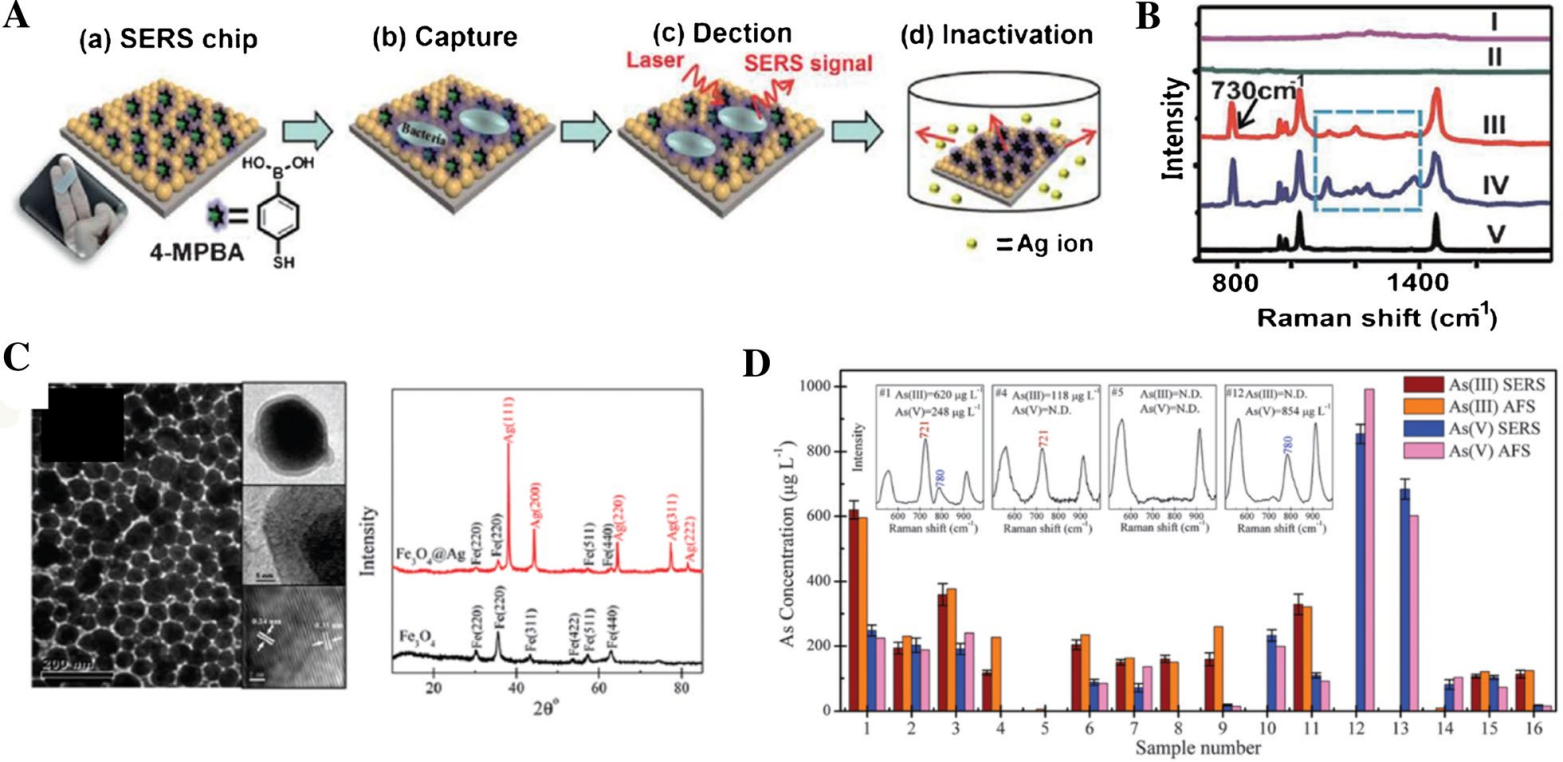
Food and environmental security tests. **A** Schematic diagram of a SERS chip prepared for bacterial detection. **B** Raman spectra obtained from bare SERS chips, prepared without 4-MPBA, and in contact with *E. coli* (*I*) or *S. aureus* (*II*), obtained from a SERS substrate treated with 4-MPBA and in contact with *E. coli* (*III*) and *S. aureus* (*IV*), or a 4-MPBA treated SERS substrate in the absence of bacteria (*V*). **C** TEM image of Fe_3_O_4_@Ag NPs and XRD measurement of Fe_3_O_4_ and Fe_3_O_4_@Ag. **D** Raman spectra of arsenite (As(III)) and arsenate (As(V)) in 12 different samples of As-contaminated groundwater. **A**, **B** Reproduced with permission from Ref. [84], © 2015, John Wiley and Sons. **C**, **D** Reproduced with permission from Ref. [86], © 2014, Royal Society of Chemistry

The detection of bacteria during the early stage of an infection can prevent severe human disease. High-sensitivity SERS substrates can detect small numbers of bacteria and prevent bacterial activation. Figure 14A shows bacteria detectable SERS substrate composed by silver nanoparticles@Si nanoparticles arrays treated with 4-mercaptophenylboronic acid (4-MPBA) on the surface. [84] Bacteria captured on the surface of a 4-MPBA treated SERS substrate will provide an enhanced Raman signal from bacteria wall due to the interaction between the bacteria and the 4-MPBA-treated silver nanoparticles. Figure 14B shows the Raman spectra obtained from SERS substrates treated with *E. coli* or *S. aureus*. The signal at 730 cm^−1^ corresponded to the Raman signal of the glycosidic bonds in the bacterial wall. The dotted box shows the Raman signal of the bacteria.

Arsenic (As) contamination in soil or groundwater causes severe health problems [85]. Among the As states, arsenite (As(III)) is much more toxic than arsenate (As(V)). The oxidation states of As should, therefore, be distinguished during environmental toxicity assays. The use of Fe_3_O_4_@Ag nanoparticles (Fig. 14C) permits the detection of As contamination [86]. Sixteen groundwater samples from different locations were quantitatively analyzed for their As contamination using the Raman spectra collected at Fe_3_O_4_@Ag nanoparticles, as shown in Fig. 14D.

### Bio-imaging and bio-sensing

**Fig. 15:**
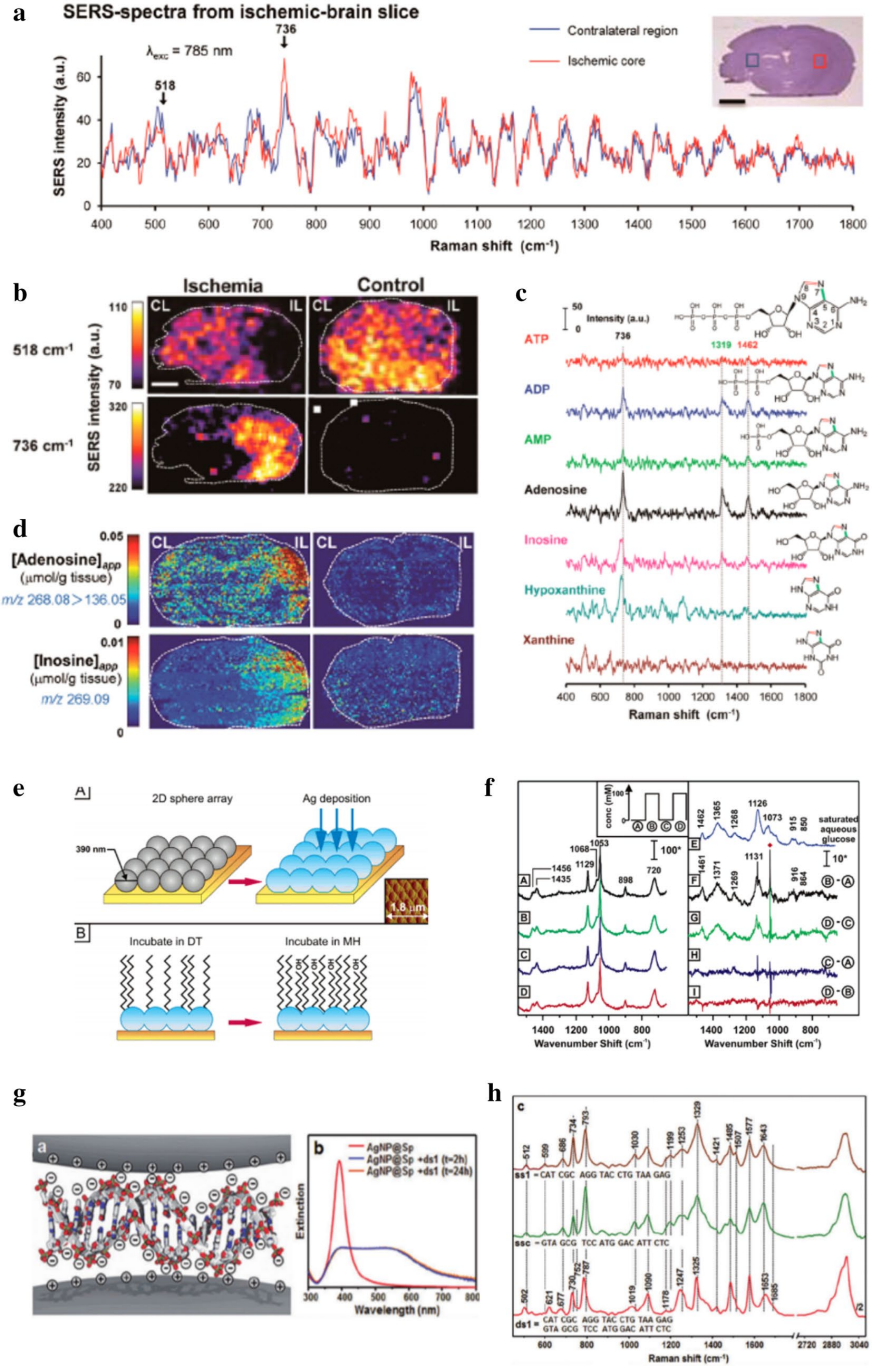
Bio-imaging and bio-sensing. **a** Raman spectra collected at two different positions of an ischemic murine brain in the core (*red*) or its contralateral region (*blue*). **b** SERS images of ischemic and control samples at 518 and 736 cm^−1^. **c** Raman spectra of eight different adenylate molecules. **d** SERS images of adenosine and inosine. **e** Schematic diagram of an AgFON substrate functionalized with a decanethiol (DT)/mercaptohexanol (MH) layer. **f** Raman spectra obtained as the glucose concentration in a solution was cycled between 0 and 100 mM (*A*–*D*). Raman spectra of a saturated aqueous glucose solution (*E*). Subtracted Raman spectrum demonstrating the adsorption/desorption of glucose from a DT/MH-AgFON substrate (*F*–*I*). **g** Schematic diagram showing a double-stranded DNA (dsDNA) molecule sandwiched within the junction between two AgNP@Sp (*left*). Extinction spectra of an AgNP@Sp suspension and AgNP@Sp with dsDNA (*right*). **h** Raman spectra of single stranded DNA (ssDNA 1), complementary ssDNA and dsDNA obtained using the AgNP@Sp substrates. **a**–**d** Reproduced with permission from Ref. [67], © 2014, American Chemical Society. **e**, **f** Reproduced with permission from Ref. [90], © 2005, American Chemical Society. **g**, **h** Reproduced with permission from Ref. [91], © 2015, John Wiley and Sons

Bio-imaging using a SERS substrate can detect dispersions of specific molecules in bio-systems [87]. Figure 15a shows the SERS spectra collected from mouse ischemic brain tissues in two different regions: an ischemic core and a contralateral region [67]. The main intensity differences were observed at 518 and 736 cm^−1^, and the intensity mappings of the mouse brains at two different Raman bands are shown in Fig. 15b. The intensity of the 518 cm^−1^ band was larger in the control region, whereas the intensity of the 736 cm^−1^ band was larger in the ischemic region. To understand which molecules were associated with ischemia, the SERS spectra of eight adenylates were measured, as shown in Fig. 15c. The SERS spectra indicated that adenosine, inosine, and hypoxanthine display large SERS intensities at 736 cm^−1^. These results verified that adenosine, inosine, and hypoxanthine molecules were associated with ischemia. Figure 15d shows the Raman mapping of adenosine and inosine onto the both ischemic and control samples.

Infinitesimal amounts of biomarkers (glucose, DNA, virus, drug) can crucially impact the viability of living organisms [88, 89]. The detection of biomarkers in a biological fluid is important for a variety of healthcare assays. For example, blood glucose levels are normally maintained between 3.9 and 5.5 mM. Diabetes mellitus patients does not regulate blood glucose levels within the normal range, and must watch these levels continuously. SERS-based glucose sensors have been fabricated using an AgFON substrate onto which has been adsorbed a decanethiol (DT)/mercaptohexanol (MH) mixture capable of trapping glucose near the surface, as shown in Fig. 15e [90]. The left-hand panel of Fig. 15f shows the SERS spectra acquired at a DT/MH modified AgFON during glucose cycling (from graphs A to E). Graphs F and G illustrate the difference between the spectra obtained at 100 or 0 mM glucose. These spectra may be compared to the spectrum obtained from a standard glucose solution (graph E), as shown in the right-hand panel of Fig. 15f. The high sensitivity of the SERS substrates to glucose molecules is apparent from these spectra. In another example, DNA analysis is important for diagnosing genetic diseases or cancers. Double-stranded DNA (dsDNA) may be selectively sandwiched between two spermine molecule-coated silver nanoparticles (AgNP@Sp) via electrostatic interactions to simultaneously provide an enhanced Raman signal from the DNA via plasmon coupling between the two neighboring silver nanoparticles, as shown in Fig. 15g [91]. The Raman signal corresponding to the dsDNA is amplified at the interparticle junctions, and the Raman spectrum of the dsDNA agrees well with that of the single-stranded sequences, as shown in Fig. 15h. This experiment demonstrated that SERS can distinguish between nucleotide sequences in DNA.

## Summary and outlook

Over the past four decades, since the discovery of SERS, a series of theoretical and technological innovations have stimulated broad interest in plasmonic nanomaterials. Advances in nanoparticle synthesis and assembly have benefited this research are. A variety of nanolithographic techniques have been developed for generating efficient SERS structures, including bottom-up lithography using colloidal templates, top-down methods using e-beam lithography, nanoimprint lithography, and laser interference lithography. From a practical point of view, simple and low-cost methods of fabricating SERS substrates are in great demand. The lithography-free hot-spot generation methods show particular promise in commercial applications, given their improvements in reproducibility and reliability.

The high sensitivity and molecular fingerprinting capabilities of the SERS technique have identified this technique as a powerful analytical tool in a wide range of chemical and biosensing applications. Practical applications of SERS technologies are under rapid development. Recent progress toward the fabrication of SERS substrates, as reviewed in this article, has been quite promising. On-site ultrasensitive SERS sensor platforms may potentially be prepared by combining SERS substrates with portable Raman spectrometers. Standard sample pretreatment protocols and the capacity for quantitatively analyzing specific target applications are needed to advance future SERS technologies.
